# A multi-spark electrohydraulic shock wave generator with adjustable pressure field distribution and beam steering capability

**DOI:** 10.3389/fruro.2023.1057723

**Published:** 2023-03-14

**Authors:** Georgy N. Sankin, Zheng Fang, Juanjuan Gu, Yun Jing, Pei Zhong

**Affiliations:** ^1^ Thomas Lord Department of Mechanical Engineering and Materials Science, Duke University, Durham, NC, United States; ^2^ Department of Mechanical and Aerospace Engineering, North Carolina State University, Raleigh, NC, United States; ^3^ Graduate Program in Acoustics, The Pennsylvania State University, University Park, PA, United States

**Keywords:** shock wave lithotripsy, electrohydraulic lithotriptor, pressure field characteristics, stone comminution, beam steering

## Abstract

**Background and objective:**

All clinical shock wave lithotripters produce an axisymmetric acoustic field without accounting for the anatomic features of the kidney or respiratory motion of the patient. This work presents a steerable and adjustable focusing electrohydraulic (SAFE) shock wave generator design with variable beam size and shape.

**Materials and methods:**

90 electrohydraulic transducers are mounted concentrically on a spherical basin with adjustable connection to individual transducers. Each transducer consists of 45 3D-printed titanium microelectrodes embedded in epoxy with a tip diameter of 0.3 mm. All the transducers are arranged in 5 concentric rings and sub-divided into 6 sectors.

**Results:**

By changing the connections of individual transducers, the focused pressure field produced by the transducer array can be either axisymmetric with a -6 dB focal width of 14.8 mm in diameter, or non-axisymmetric with a long axis of 22.7 mm and a short axis of 15.1 mm. The elongated beam produces a peak positive pressure of 33.7 ± 4.1 MPa and comminution efficiency of 42.2 ± 3.5%, compared to 36.2 ± 0.7 MPa and 28.6 ± 6.1% for axisymmetric beam after 150 pulses at 20 kV.

**Conclusions:**

We have demonstrated that the SAFE shock wave generator can produce an elongated non-axisymmetric pressure field with higher stone comminution efficiency. The SAFE shock wave generator may provide a flexible and versatile design to achieve accurate, stable, and safe lithotripsy for kidney stone treatment.

## Introduction

Kidney stone disease (or urolithiasis) affects 5-15% of the population worldwide (1). In the United States, shock wave lithotripsy (SWL) is still used as the first line therapy for about 50% of the stone patients in spite of the technical advances and growing popularity of ureteroscopy in the past decade (2). During clinical SWL, however, elevated dose delivered at fast rate is often preferred, in association with the high pressure and pulse energy generated by the contemporary lithotripters, which increases the risk of renal injury, i.e., hemorrhage and perinephric hematomas (3–6). It is therefore highly desirable to reduce the total number of high energy shock waves delivered to the patient to minimize the risk of tissue injury while maintaining successful stone comminution in SWL.

Two primary factors have been identified that contribute to the elevated shock wave exposure during SWL: 1) patient’s respiratory motion and 2) residual fragment dispersion (7, 8). Using B–mode ultrasound, Sorensen et al. have shown that kidney stones may move by 10-40 mm and ureteral stones by 7-10 mm in patients under sedation during SWL, leading to about 40% of the lithotripter-generated shock waves (LSWs) missing the target stones (7). In addition, following the initial breakup, residual fragments may spread out inside the renal collecting system to an area exceeding the fragmentation zone of the lithotripter, rendering the ensuing shock waves less effective (8).

The respiration trajectory of the kidney is roughly aligned in parallel to the spine along the head-to-toe direction of the patient. In contrast, the spreading of residual fragments will depend on the anatomic features of the kidney (i.e., renal calyces and pelvis), or the orientation of the ureter where the stone resides. Matching the pressure or energy distribution with these anatomic features or respiratory motion patterns in stone patients during SWL are difficult using contemporary clinical lithotripters because they can only generate axisymmetric pressure fields along the LSW propagation direction. On the other hand, it is conceivable that an optimized distribution of the shock wave energy to maximally match with the stone or fragments trajectory during SWL will improve treatment efficiency while minimizing tissue injury.

We have previously used a foam mask placed above the acoustic lens of an electromagnetic shock wave source to transform its pressure distribution in the focal plane from an axisymmetric to an elongated non-axisymmetric field (9, 10). We have shown *in vitro* that by matching the pattern of respiratory stone-movement in patient, 30% improvement in targeting accuracy can be achieved (9). The mask design, however, cannot be used easily for flexible re-configuration of the acoustic field to track a moving stone target.

In this study, we describe the development of a prototype steerable and adjustable focusing electrohydraulic (SAFE) shock wave generator for flexible lithotripter field control utilizing a re-configurable multi-spark transducer array. We present the design principle, preliminary tests of acoustic field characterization and *in vitro* stone comminution, demonstrating several unique advantages of this new shock wave source that may provide flexible beam forming and stone targeting capability during clinical SWL procedures.

## Materials and methods

### Design concept

**Figure 1:**
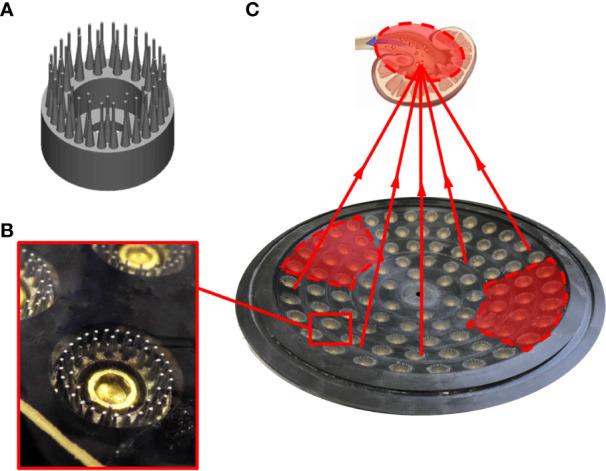
**(A)** CAD model of the 45-pin transducer; **(B)** enlarged photo of the 45-pin transducer on the SAFE; **(C)** illustration of wave propagated from the SAFE and focus at kidney stones, with two side sections in red shaded areas disconnected, leading to a focal zone elongation in the side-section direction.

In the SAFE shock wave generator, 90 individual electrohydraulic transducers are mounted on a spherical carrier made of dielectric material. Each transducer consists of 45 3D-printed titanium electrodes embedded in epoxy resin and each polished to yield a tip diameter of 0.3 mm following previous studies (11, 12). The transducers are arranged in 5 concentric rings divided into 6 sectors (**Figure 1**).

**Figure 2:**
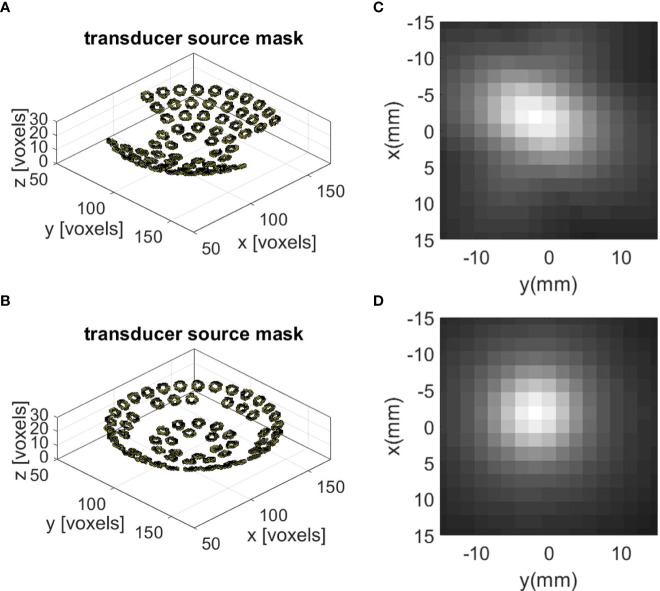
Schematic view of the transducers distribution on the spherical carrier and resulting pressure contour in the focal plane in **(A)** and **(C)** Case A, and **(B)** and **(D)** Case B.

In linear acoustics, the beam width is inversely proportional to the aperture diameter of the acoustic source (13). Therefore, for a given focal length, by reducing the active section dimension of the shock wave generator, for example along a 45-degree angle with respect to the y-axis in **Figure 2**, we can effectively increase the corresponding beam width in a particular direction. This design strategy is used to transform an axisymmetric pressure field into a non-axisymmetric, elongated focal zone (**Figure 2**).

The numerical simulations throughout this pilot study were carried out using an open-source toolbox k-Wave (14) in time-domain under linear wave propagation assumption. While the peak pressure may be underestimated, the linear wave propagation model is anticipated to qualitatively capturing the focal pressure distribution for the purpose of comparison between the axisymmetric vs. elongated pressure fields. In the simulation, the voxel size was 1.875 mm, whereas the time step size was 0.156 microseconds. The excitation waveform was taken from Eq. 8 in (15). More realistic waveform generated by micro-spark at individual pin tip will be used in future simulations.

Experiments were conducted using two different beam configurations, designed for employing the same total number of activated pins (*N_p_*) yet with different transducer distributions in the SAFE shock wave generator. To produce a non-axisymmetric or elongated beam (Case A), 12 transducers on the two opposite sectors (12x2 = 24) were disconnected (**Figure 1C**). In contrast, to produce an axisymmetric beam (Case B), all 4 transducers in the third ring for each of the six sectors (6x4 = 24) were disconnected (see **Figure 2**). Hence, the total number of transducers (*N_t_*) where *N_t_* = 90 - 24 = 66 were activated in both case A and case B. If we assume that the total input electric energy [*E_total_* = CU^2^/2 = 0.5×3μF×(15 kV)^2^ = 0.34 kJ] was evenly distributed to *N_p_* activated pins, the electric energy delivered to each activated pin (*E_p_*) can then be calculated by:


(1) $$E_{p}=\frac{E_{total}}{N_{p}}=\frac{\frac{1}{2}CU^{2}}{N_{p}}=0.11\;J,$$

where *N_p_* = *N_t_* × 45 = 2970, *C* = 3 µF is the capacitance, and *U* is the charging voltage.

### Acoustic field characterization

The SAFE shock wave generator, installed at the bottom of a cylindrical acrylic water tank (Ø254×H305 mm), was triggered by a digital delay pulse generator (BNC Model 555, Berkley Nucleonics). A metal mesh with grid size 12.5×12.5 mm was mounted above the surface of the titanium electrodes and grounded. The tank was filled with electrolyte (1% sodium chloride in water) to ensure synchronization of the spark discharges from all transducer tips. A fiber optic probe hydrophone (FOPH 500, RP Acoustics, Leutenbach, Germany) mounted on a computer-controlled 3D translational stage (VXM-2 step motors with BiSlide-M02 lead screw, Velmex, Bloomfield, NY) was used for scanning and pressure measurements in the shock wave focal plane.

The pressure measurements were performed at U = 15 kV for beam configuration comparison. In addition, pressure was measured at the beam focus under *U* = 20 kV, which was used for stone comminution experiments. The pressure measurements were repeated at least three times at each point.

### Stone fragmentation assessment

**Figure 3:**
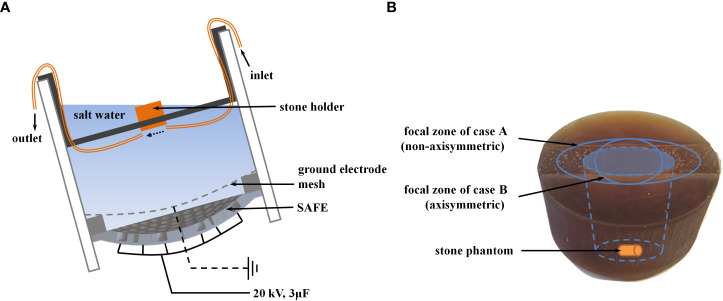
**(A)** Photo of the stone holder with the illustration of stone phantom location; **(B)** diagram of the experimental setup for stone fragmentation test.

Stone fragmentation experiments were performed using cylindrical soft BegoStone (5:2 powder to water mixing ratio) of Ø6×H6 mm in size and 0.33 g in weight (16). Stone phantoms were soaked in water for more than 30 minutes before the fragmentation test. As shown in **Figure 3**, a polyurethane rubber stone holder (Ø48mm outer diameter, 30 mm height) in elliptical shape (long axis: 24 mm, and short axis: 12 mm) was used to approximate the anatomic geometry of the renal pelvis while allowing residual stone fragments to be dispersed laterally during SWL.

A water circulation system was constructed to facilitate removal of bubble remnants accumulated underneath the stone holder during SWL (17). The pre-soaked stone sample was placed inside the polyurethane holder and aligned with the focus of the SAFE shock wave generator. After treatment with 150 pulses produced at 20 kV, stone fragments were collected and air dried overnight. Afterwards, fragments were filtered through 2 mm and 2.8 mm grid sieves (W.S. Tyler, Mentor, OH), and weighted to calculate the stone comminution efficiency based on the percentage of the residual fragments over the original stone weight. Six stones were treated for each case. Data were post-processed in Excel (Microsoft, Redmond, WA) and presented in bar chart with mean, standard deviation, and p-value.

## Results and discussion

**Figure 4:**
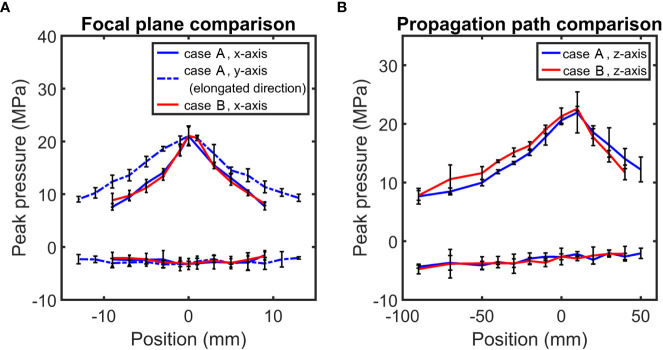
Peak positive pressure (*p+*) and peak negative pressure (p-) distribution comparison on **(A)** focal plane (x- and y-axis), and **(B)** along wave propagation path (z-axis) between Case A and Case B.

**Figure 4A** shows the peak pressure (*p+*) distribution along two orthogonal directions in the focal plane (z = 0 mm). The -6 dB focal width, estimated by the full width at half maximum using a Gaussian curve fitting for *p+*, was found to be 22.7 mm (along the side-section direction) by 15.1 mm (in the orthogonal direction) for the elongated beam. In comparison, the -6 dB focal width of the axisymmetric focal zone was about 14.8 mm in both directions. Moreover, the pressure distributions along the z-axis were found to be comparable between the two configurations (**Figure 4B**). The slope of pressure change post-focally (i.e., z ≥ 0) is steeper than its counterpart pre-focally (i.e., z ≤ 0). These results are summarized in **Table 1**. In comparison, based on linear wave model simulation, the -6 dB focal width is 18.5 mm along the side-section direction (Case A, non-axisymmetric, **Figure 2**) by 11.5 mm. In contrast, the -6 dB focal width of the axisymmetric focal zone (Case B, axisymmetric, **Figure 2**) is 13.0 mm along both directions. In general, the trends in the focal width change of the shock wave generator between experimental measurements and model simulations are similar, with an average discrepancy about 20%. These discrepancies are likely to be reduced when the nonlinear wave propagation is included in the future modeling work.

**Table 1:** Characteristics comparison of the SAFE in different cases.

Case	*N_t_*	Transducer geometry	Experiment *p+* (MPa)	Experiment *FW_x_* (mm)	Experiment *FW_y_* (mm)	Simulation *FW_x_* (mm)	Simulation *FW_y_* (mm)
A	66	non-axisymmetric	21.1	15.1	22.7	11.5	18.5
B	66	axisymmetric	21.8	14.8	14.8	13.0	13.0

N_t_ is the number of activated transducers.

**Figure 5:**
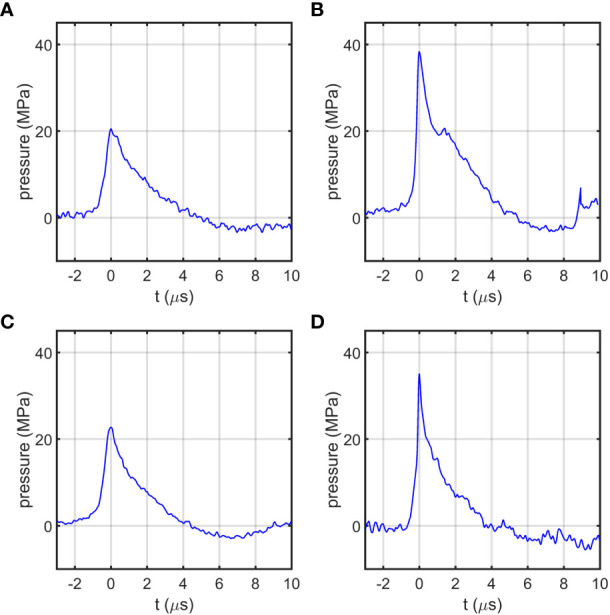
Pressure waveforms measured at focal point when input voltage U = 15 kV and 20 kV in **(A)** and **(B)** Case A, and **(C)** and **(D)** Case B.

As the input voltage increases from 15 kV to 20 kV (with the corresponding total input electric energy varying from 338 J to 600 J), the value of *p+* increases from 21.1 ± 1.1 MPa to 33.7 ± 4.1 MPa for the elongated beam. Similarly, *p+* increases from 21.8 ± 0.8 MPa to 36.2 ± 0.7 MPa for the axisymmetric beam (**Figure 5**). At 20 kV, the rise time of the shock wavefront is in the range of 0.2 μs to 0.3 μs (**Table 2**). These acoustic field parameters of the SAFE shock wave generator are comparable to the corresponding values in an HM3 lithotripter, except the longer rise time of the shock front (see **Table 2**). In comparison to the HM3, the SAFE shock wave generator has the unique advantage of transforming the axisymmetric acoustic field in the lithotripter focal plane to an elongated (oval shape) pressure field that can better match with the anatomical features of the kidney and/or the trajectory of respiratory motion of the patients during SWL. More importantly, the SAFE shock wave generator has the potential of flexible control of the lithotripter focal beam size, shape, and orientations.

**Table 2:** Comparison of the acoustic fields and stone comminution (SC) efficiency produced by the SAFE and Dornier HM3.

		Working voltage U(kV)	Working capacity C(µF)	N_t_	E_p_ (J/pin)	p_+_ (MPa)	p_-_ (MPa)	t_r_ (µs)	t_+_ (µs)	t_-_ (µs)	Stone comminution (<2mm)
SAFE	Case A	20	3	66	0.202	33.7 ± 4.1	-4.3	0.24	~5.5	~6.0	42% (150 pulses)
	Case B			66	0.202	36.2 ± 0.7	-5.5	0.31	~4.5	~7.0	29% (150 pulses)
HM3		20	0.08	1	16	48.9	-8.0	<0.030	1~2	4~6	<30% (150 pulses in the membrane holder)

Data compiled from (18–22) and this study.

**Figure 6:**
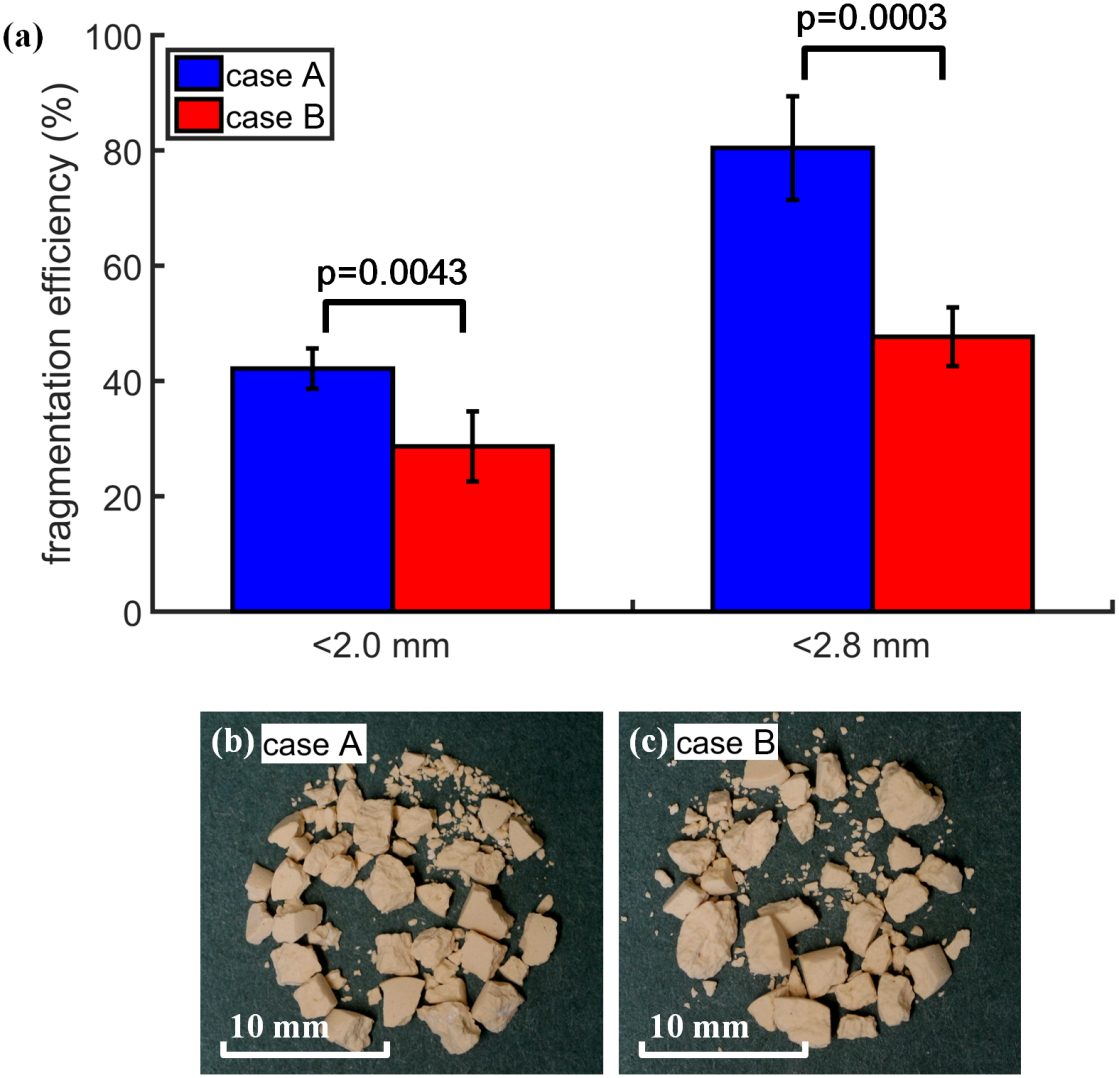
**(A)** Stone comminution (n = 5) of fragments smaller than 2 mm (left) and 2.8 mm (right) after 150 pulses of shock wave treatment by the SAFE in case A (blue) and case B (red). Indicated p-values smaller than 0.05 for each group of results. Stone fragments after 150 pulses of shock wave treatment by the SAFE in both **(B)** Case A and **(C)** Case B.

A total of 12 stones were treated. However, one outlier in each group was detected and removed, resulting in a final sample size of n = 5 (**Figure 6A**). For the elongated beam, stone fragmentation rate for fragments less than 2.0 mm was 42.2 ± 3.5% compared to 28.6 ± 6.1% for the axisymmetric beam. For stone fragments smaller than 2.8 mm, stone comminution for the elongated beam (Case A: 80% ± 9%) is greater than that of the axisymmetric beam (Case B: 47.7% ± 5.1%). Two-tail t-test of the data from the two groups show p-values of 0.0043 (<2 mm) and 0.0003 for (<2.8 mm), respectively, indicating statistically significant difference in stone fragmentation produced by the two configurations of the SAFE shock wave generator. Graphically, **Figures 6B, C** display the stone fragments after SWL treatment. Stones treated by the elongated beam have more fragments and smaller sizes compared to those produced by the axisymmetric beam. These results suggest that higher stone comminution efficiency may be produced by adjusting the beam size and shape to better match with the target stone/fragments trajectory during clinical SWL procedures.

## Conclusion

In this study, we present the prototype design and evaluation of a steerable and adjustable focusing electrohydraulic (SAFE) shock wave generator. Such a design provides beam-forming flexibility in SWL, which allows us to better match the acoustic field of the lithotripter with anatomic features and spreading of residual fragments in stone patients. Improved stone comminution efficiency has been demonstrated in an elliptical stone holder. Future work is warranted in electronic control of beam forming and steering, evaluation of safety and treatment efficiency in animal models. Pressure measurements and model simulation along the direction of the shock wave propagation will also be conducted.

## Data availability statement

The original contributions presented in the study are included in the article/supplementary material. Further inquiries can be directed to the corresponding author.

## Author contributions

GS designed and constructed the multi-spark shock wave generator, and together with ZF carried out the pressure measurement and stone comminution tests. YJ performed numerical model simulation of the axisymmetric and non-axisymmetric pressure field with technical assistance of JG. PZ supervised the development of the study plan, data analysis and preparation of the manuscript. All authors contributed to the article and approved the submitted version.
